# Assessment of Type 1 Diabetes Risk Conferred by HLA-DRB1, INS-VNTR and PTPN22 Genes Using the Bayesian Network Approach

**DOI:** 10.1371/journal.pone.0079506

**Published:** 2013-11-18

**Authors:** Rosalba Portuesi, Paolo Pozzilli, Bernhard Boehm, Raffaella Buzzetti, Simonetta Filippi

**Affiliations:** 1 Department of Endocrinology and Diabetes, University Campus Bio-Medico, Rome, Italy; 2 Department of Gynecology, University Campus Bio-Medico, Rome, Italy; 3 Blizard Institute, Center of Diabetes, St Bartholomew’s and the London School of Medicine, Queen Mary, University of London, London, United Kingdom; 4 Division of Endocrinology and Diabetes, Ulm University, Ulm, Germany; 5 Department of Experimental Medicine, University of Rome “Sapienza”, Rome, Italy; 6 Laboratory of Non Linear Physics and Mathematical Models, University Campus Bio-Medico, Rome, Italy; Children’s Hospital Boston/Harvard Medical School, United States of America

## Abstract

**Background:**

Determining genetic risk is a fundamental prerequisite for the implementation of primary prevention trials for type 1 diabetes (T1D). The aim of this study was to assess the risk conferred by HLA-DRB1, INS-VNTR and PTPN22 single genes on the onset of T1D and the joint risk conferred by all these three susceptibility loci using the Bayesian Network (BN) approach in both population-based case-control and family clustering data sets.

**Methodology/Principal Findings:**

A case-control French cohort, consisting of 868 T1D patients and 73 French control subjects, a French family data set consisting of 1694 T1D patients and 2340 controls were analysed. We studied both samples separately applying the BN probabilistic approach, that is a graphical model that encodes probabilistic relationships among variables of interest. As expected HLA-DRB1 is the most relevant susceptibility gene. We proved that INS and PTPN22 genes marginally influence T1D risk in all risk HLA-DRB1 genotype categories. The absolute risk conferred by carrying simultaneously high, moderate or low risk HLA-DRB1 genotypes together with at risk INS and PTPN22 genotypes, was 11.5%, 1.7% and 0.1% in the case-control sample and 19.8%, 6.6% and 2.2% in the family cohort, respectively.

**Conclusions/Significance:**

This work represents, to the best of our knowledge, the first study based on both case-control and family data sets, showing the joint effect of HLA, INS and PTPN22 in a T1D Caucasian population with a wide range of age at T1D onset, adding new insights to previous findings regarding data sets consisting of patients and controls <15 years at onset.

## Introduction

The incidence of type 1 diabetes (T1D) has been increasing worldwide by approximately 3% per year, with the highest increase occurring in young children [1]–[3]. Namely, incidence has increased with a rise of 5.3% in North America, 4% in Asia, and 3.2% in Europe [4], [5]. It is well known that the major T1D susceptibility locus maps to the class II loci HLA-DRB1 and HLA-DQB1 on chromosome 6p21. The highest risk DR/DQ haplotypes for T1D are DR3-DQA1*0501-DQB1*0201 (DR3) and DR 4-DQA1*0301-DQB1*0302 (DR4), and accounts for up to 30%–50% of the genetic T1D risk [6]. Other non-HLA T1D loci as the INS gene [7], the CTLA4 gene [8], the PTPN22 gene [9] together with other susceptibility genes have smaller effects on disease risk [10]. The INS gene located on chromosome 11p15.5 confers about 10% of the genetic susceptibility to T1D. The variable number of tandem repeats located 0.5 kb upstream of INS [11] and other polymorphisms in tight linkage disequilibrium such as 23HphI and 1140A/C [12] have been implicated as etiological factor in T1D. The PTPN22 gene is located on chromosome 1p13 and encodes a lymphoid protein tyrosine phosphatase (LYP) that is important in negative control of T-cell activation and in T-cell development.

The pattern of inheritance in T1D is complex and the development of the disease is thought to be determined by an interaction between genetic predisposition and environmental triggers. There is a high familial clustering with a mean prevalence of 6% in siblings compared to 0.4% in Caucasian populations, although more than 85% of patients with T1D lack a positive family history for the disease [13]. The two primary approaches used to identify risk loci for T1D have been linkage studies and association studies. Linkage studies, typically using affected sibling pairs, can identify regions of the genome that are shared more frequently among affected relatives [14].

In contrast to linkage studies, association studies can detect alleles with much more modest effects on risk as long as those alleles are relatively common. All of the four well-established risk loci, together including HLA, INS, CTLA4, and PTPN22, were identified in candidate gene association studies.

The risk can be further stratified by recruitment of subjects with susceptible genotypes in a case-control study and by selection of children with a multiple family history of diabetes by collecting genetic data of a family cluster.

The aim of the present study was to assess the risk conferred by HLA-DRB1, INS-VNTR and PTPN22 loci on the onset of T1D and the joint risk conferred by all these three susceptibility loci, using the Bayesian Network (BN) approach in both population-based case-control and family clustering data sets [15].

## Materials and Methods

### Data Sets

The case-control French cohort (data set A) consisted of 868 French Caucasian T1D patients (M/F 0.84, mean age at T1D onset 19.63*±*14.40 years) and 73 French Caucasians control subjects (0.63 M/F ratio), recruited in three hospitals in Paris and Lille [16].

French nuclear family’s data set (data set B) included 1694 patients (M/F 1.2, mean age at T1D onset 14.5±10.3) and 2340 controls (M/F 0.88). Only two phenotypes, with or without the disease, and no intermediate phenotypes such as those positive for islet cell autoantibodies and impaired glucose tolerance, were investigated. Data sets used in our analysis consisted of less data than the original ones because we excluded individuals with missing genotypes.

### Gene Typing

All individuals taking part in these studies gave their informed consent for genetic studies. DNA was extracted from blood using standard techniques and genotyping for HLA-DRB1, INS VNTR and PTPN22 genes was performed [16]–[17]. Genotyping of HLA-DRB1 alleles (DR3 and DR4) was performed using a PCR amplification with sequence-specific primers (PCR-SSP) in 2 hours. PCR-SSP is an accurate typing technique with high sensitivity, specificity and reproducibility. DR “low-resolution” typing by the PCR-SSP technique is ideally suited for analyzing small numbers of samples simultaneously [18].

To determine the susceptibility status at the INS (IDDM2) locus, the INS polymorphism INS-23/HphI was genotyped [19].

Genotyping of PTPN22 C1858T (R620W, rs2476601) was performed using a TaqMan assay (Applied Biosystems), or a PCR–restriction fragment–length polymorphism assay, with identical results [20].

### Gene Risk Classification

All genetic information was classified based on T1D susceptibility degree risk, as follows:

#### HLA

Subjects were grouped from the highest (DRB1*03/DRB1*04) to moderate (DRB1*03/DRB1*03 or DRB1*04/DRB1*04; DRB1*03/DRX or DRB1*04/DRX) to the lowest (DRB1*X/DRB1*X, where X is other than DRB1*03 or DRB1*04 allele) HLA genotypes for T1D risk [21].

### INS Gene

The short class I variable number of tandem repeats alleles were associated with predisposition to T1D, whereas class III alleles were dominantly protective. So that, A/T and T/T were considered as the non-susceptibility genotypes and AA as the susceptibility genotype [22].“INS” node represented in the BN graph, included both risk classes.

### PTPN22 Gene

We examined and compare the allelic effect of T (variant allele) vs. C (common allele), in terms of contrast of T/T+T/C vs C/C genotypes. Namely, C/C was considered as the non-susceptibility genotype whereas T/T and C/T as susceptibility genotypes [9], [23].

We analyzed both samples separately using the BNs approach.

### Bayesian Network (BN) Approach

**Figure 1:**
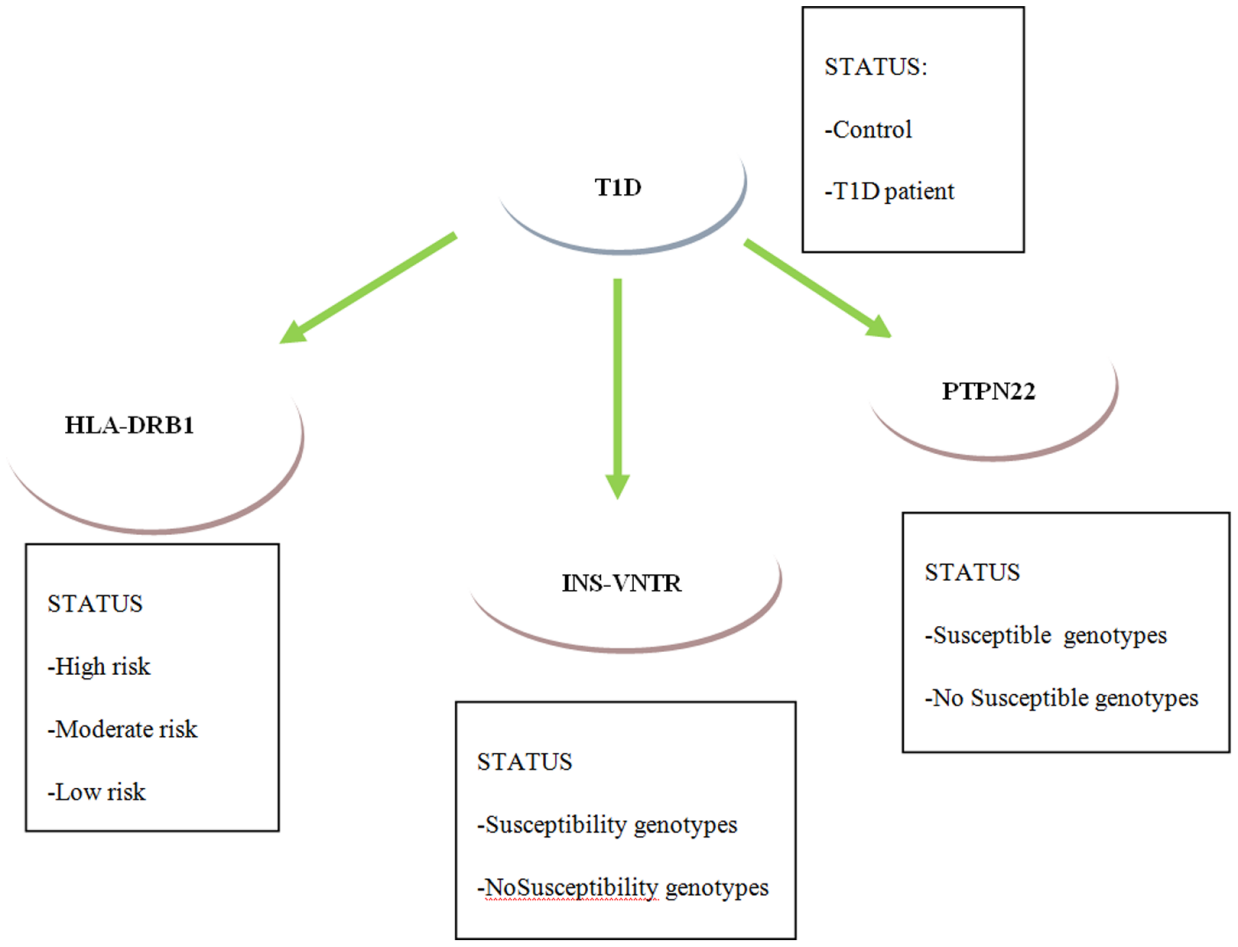
The Bayesian Network implemented to assess risk to develop T1D.

BN provides a powerful and flexible tool for reasoning under uncertain conditions [24]–[27]. BN is a graphical model that encodes probabilistic relationships among variables of interest. Each variable is represented graphically by a node and the links (edges) between nodes correspond to the probabilistic dependence between variables. Furthermore, each node has a conditional probability table, quantifying the relationship between connected variables. It is possible to set the values of any combination of nodes in the network and this evidence propagates through the network producing a new probability distribution over all the variables in the network. In our graph (Fig.1) HLA-DRB1, INS-VNTR and PTPN22 gene variables are denoted as nodes. Each node may have a set of states corresponding to risk classes (high, moderate and low risk for HLA, susceptibility/non-susceptibility for INS-VNTR and PTPN22). T1D node consisted of 2 states: normal and subjects.

Associated with an arrow linking two nodes, a conditional probability table exists that estimates the value of the likelihood of the state of the second gene given the state of the first gene. Bayesian approach makes it possible to systematically integrate experimental data with multiple sources of “prior” knowledge (called “prior” value), as the existing large body of published literature. In our case the prior value was the prevalence of T1D in French population, which is 0.4% [13]. When the family data was analyzed, a prevalence value of 6% was considered as reported in literature for risk for T1D in siblings [13]. Prevalence value varies between different populations around the world and with it also changes the risk conferred by risk factors. For this reason introducing the right prior information is crucial in the evaluation of risk factors.

After introducing prevalence value, BN algorithm updated automatically population composition and with it also the percentage of the three gene risk classes was adjusted accordingly (e.g. percentage of high, moderate and low risk classes for HLA were 32%, 55%, 12% before entering the prevalence value, and 2.8%, 34.3% and 62.7% after introducing it, respectively). Following the process of learning from data implemented with the R program, BN was questioned on T1D genetic risk conferred by all single susceptibility genes but each one separately in both allelic risk statuses. Moreover, for all possible status combinations of the three genes, a risk value was calculated and the best network fitting data was selected using AIC score and P-value.

### Significance: P-Value and AIC Score

The measure of the quality of a BN can be computed using several scores. The goal of the score is to figure out how accurately models will predict new data when fitted to the old ones. Here AIC score (Akaike Information Criterion) [28] was chosen. AIC score, as an estimator of predictive accuracy, figures out how accurately BN models define the relationship between nodes. According to this criterion the network with the highest AIC score was selected as the best network.

The validity of the edges can be measured by testing the mutual information between a parent node and the corresponding child. The mutual information can then be compared to a chi-square distribution. The corresponding P-value can be seen as the strength of the edge and so of the relationship between the nodes.

## Results

### Case-control Study (Data Set A)

#### Genes – T1D correlation

When the relationship between HLA-DRB1, INS and PTPN22 genotypes was evaluated separately, a significant association between these genes and T1D was found (p = 0.003 for HLA, p = 0.9 *10^−3^ for INS gene and p = 1.5*10^−5^ for PTPN22).

### Single-locus Analysis

#### HLA-DRB1 locus

When HLA-DRB1 genotypes were considered, the risk values of developing T1D were 4.8%, 0.6% and 0.05% for high, moderate and low risk HLA-DRB1 genotypes, respectively.

#### INS locus

When INS genotypes were considered, the risk values of developing T1D were 0.6% and 0.19% for at risk and non at risk INS genotypes, respectively.

#### PTPN22 locus

When PTPN22 genotypes were considered, the risk values of developing T1D were 0.6% and 0.35% for at risk and not at risk PTPN22 genotypes, respectively.

In Table 1 the risk analysis using OR (Odds Ratio) parameter and BN algorithm to evaluate single-locus main effects are reported.

**Table 1:** Risk analysis using OR parameter and BN algorithm to evaluate single-locus main effects (Case-Control study - Data set A).

Genes	Cases(N)	Controls(N)	OR	95%CI	BN risk(%)
**HLA**					
High risk	300	2	18.7	4.5–76.9	**4.8**
Moderaterisk	494	25	2.51	1.5–4.1	**0.6**
Low risk	74	46	0.05	0.03–0.09	**0.05**
Total	868	73			
**INS**					
Risk	640	33	3.4	2.09–5.52	**0.65**
No risk	228	40	1		**0.19**
Total	868	73			
**PTPN22**					
Risk	260	12	2.1	1.1–4.1	**0.6**
No risk	608	61	1		**0.35**
Total	868	73			

#### Joint effect of HLA and INS loci

We found significant heterogeneity in the distribution of the INS genotypes (susceptibility/non-susceptibility) in the three HLA risk categories when the patients’ group was considered, in agreement with data from literature [30].

The analysis of the heterogeneity showed that the INS predisposing genotype was more common in both moderate and low-risk HLA-DRB1 genotype patients (77.3%and 71.6%, respectively), than in patients carrying the high risk HLA-DRB1 genotype (68.3%) (Chi-square = 7.98, degrees of freedom = 2, p = 0.01). No evidence of heterogeneity was observed in the distribution of the INS genotypes in the control group where the INS genotypes were similarly represented within the different HLA risk categories (p = 0.2).

The joint risk to become T1D for a subject with high, moderate or low HLA-DRB1 risk genotypes in association with at risk or not at risk INS genotypes, was calculated using the BN algorithm.

Considering the at risk or not at risk, INS genotypes, the absolute risk were 7.6% and 2.3% respectively in high risk HLA-DRB1 subjects; 1.07% and 0.3% in moderate risk HLADRB1 subjects; and 0.08% and 0.02% in low risk HLA-DRB1 subjects.

#### Joint effect of HLA and PTPN22 loci

The joint effect of PTPN22 and HLA varied across the HLA risk categories. Considering at risk and not at risk PTPN22 genotypes, the absolute risks were 7% and 4.2% respectively in HLA-DRB1 high-risk subjects; 1% and 0.7% in HLA-DRB1 moderate-risk subjects and 0.08% and 0.05% in HLA-DRB1 low-risk subjects.

#### Joint effect of INS and PTPN22 loci

When INS and PTPN22 risk genotypes were evaluated to establish the risk for T1D onset, the risk values were equal to 1% for subjects with both INS and PTPN22 risk genotypes. The risk decreased to 0.15% when non at risk genotypes were present at both loci.

#### Joint effect of HLA, INS and PTPN22 loci

We also tested models with all three-way interactions involving the three susceptibility loci. Results showed that more risk loci an individual carries, higher is the absolute risk, as expected; the presence or absence of HLA risk loci influences the absolute risk much more than the other loci. For instance, carrying risk genotypes at INS VNTR and PTPN22 but not at the HLA-DRB1 locus is associated with a much lower risk compared to the condition in which HLA-DRB1 high risk genotypes are with low-risk genotypes at the other two loci. The absolute risks conferred by simultaneously carrying high, moderate or low-risk at HLA-DRB1 locus and all risk genotypes at the other two loci, were 11.5%, 1.7% and 0.1%, respectively (Table 2).

**Table 2:** Distribution of the different risk genotypes at the three susceptibility loci among cases and controls (Data set A).

HLAclasses	INS^a^	PTPN^a^	Cases	Controls	OR(95%CI)^b^	BNrisk
High	R	R	51	0	108(6.2–189)	11.5
High	R	NR	154	2	81(17–377)	7
High	NR	R	32	0	68(4–1201)	3
High	NR	NR	319	0	673(39–11597)	1.8
Moderate	R	R	118	1	124(15–988)	1.7
Moderate	R	NR	264	10	27(11.3–68)	1
Moderate	NR	R	38	2	20(4.2–95)	0.4
Moderate	NR	NR	74	12	6.5(2.6–15)	0.24
Low	R	R	18	2	9.5(1.9–46)	0.1
Low	R	NR	35	18	2.05(0.8–4.8)	0.08
Low	NR	R	3	7	0.4(0.1–2)	0.03
Low(reference)	NR	NR	18	19	1	0.02

a “R”genotype at given locus associated with risk; “NR”, genotype at given locus not associated with risk;

b Odds ratio vs single reference group without risk genotype at any of the four loci.

### Family Study (Data Set B)

#### Genes – T1D correlation

When the relationship between HLA, INS and PTPN22 genotypes was evaluated separately, a significant association between genes and T1D was found (p = 0.01 for HLA, p = 1*10^−3^ for INS gene and p = 3*10^−4^ for PTPN22).

### Single-locus Analysis

#### HLA-DRB1 locus

Compared with the T1D absolute risk of 6% in Caucasian siblings, the HLA-DRB1 high risk genotypes conferred a risk of 15.9%. For moderate and low risk categories the risk of developing T1D was 5.1% and 1.68% respectively.

#### INS locus

When INS genotypes were considered, the risk values of being T1D were 6.6% and 4.7% for at risk and not at risk genotypes.

#### PTPN22 locus

When PTPN22 genotypes were considered, the risk values of being T1D were 6.9% and 5.6% for at risk and not at risk genotypes, PTPN22 respectively.

#### Joint effect of HLA and INS loci

We did not find a significant heterogeneity in the distribution of the INS genotypes (susceptibility/non-susceptibility) in HLA-DRB1 risk categories when patients and controls were considered (patients group: chi-square = 3.80, degrees of freedom = 2, p = 0.149; controls group: chi-square = 0.9, degrees of freedom = 2, p = 0.64, NS).

The joint risk values to have T1D in a subject with high, moderate or low risk HLA-DRB1 categories, in association with at risk or not at risk INS genotypes, were calculated using BN algorithm. Our results showed that the relative impact of variation at INS locus was evident in all different HLA-DRB1 genotype categories. The absolute risk was of 17.3% and 12.7% if INS at risk or no at risk genotypes were respectively present in individuals with high-risk HLA-DRB1, 5.7% and 4% in individuals with moderate-risk HLA-DRB1, and 1.8% and 1.3% in individuals with low-risk HLA-DRB1 genotypes (Table 3).

**Table 3:** Risk analysis using OR parameter and BN algorithm to evaluate single-locus main effects (Family study - Data set B).

Genes	Cases(N)	Controls(N)	OR	95% CI	BN risk(%)
**HLA**					
High risk	620	289	4.1	3.4–4.7	**15.9**
Moderate risk	987	1604	0.6	0.5–0.7	**5.1**
Low risk	87	447	0.04	0.03–0.05	**1.68**
Total	1694	2340			
**INS**					
Risk	1267	1577	1.4	1.2–1.6	**6.6**
No risk	427	763	1		**4.7**
Total	1694	2340			
**PTPN22**					
Risk	550	645	1.2	1.1–1.4	**6.9**
No risk	1144	1695	1		**5.6**
Total	1694	2340			

#### Joint effect of HLA and PTPN22 loci

Our results showed that the relative impact of variation at PTPN22 was evident in all HLA-DRB1 genotype categories. The absolute risk of 18.2% and 14.9% if PTPN22 genotypes were at risk or no at risk, respectively, was detected in individuals with high-risk HLA-DRB1, 6% and 4.8% in individuals with moderate-risk HLA-DRB1 and 1.9% and 1.5% in individuals with low-risk HLA-DRB1 genotypes.

#### Joint effect of INS and PTPN22 loci

When INS and PTPN22 risk genotypes were evaluated to establish the risk of being T1D, the risk values were equal to 7.7% for subjects with both INS and PTPN22 risk genotypes. The risk decreased to 4.3% when both these genes were present with non at risk genotypes.

#### Joint effect of HLA, INS and PTPN22 loci

We also tested models with all three-way interactions involving the three susceptibility loci. The results showed that the more risk loci an individual carries, higher is the absolute risk (19.8%), but, as expected, the presence or absence of HLA-DRB1 risk genotype influences the absolute risk much more than the other loci. Carrying risk genotypes at both INS and PTPN22 but not at HLA-DRB1 locus is associated with a much lower risk compared to the presence of HLA-DRB1 risk genotypes with low-risk genotypes at INS and PTPN22 loci. The absolute risk (BN risk) conferred by simultaneously carrying high, moderate or low risk HLA-DRB1 and risk genotypes at the other two loci, at all three loci, was 19.8%, 6.6% and 2.2%, respectively (Table 4).

**Table 4:** Distribution of the different risk genotypes at the three susceptibility loci (Family study – Data set B).

HLAclasses	INS^a^	PTPN^a^	Cases	Controls	OR(95%CI) b	BNrisk
High	R	R	145	50	23.4(12–45)	19.8
High	R	NR	303	138	17.7(9.6–32)	16.3
High	NR	R	52	29	14.5(6.9–30)	14.7
High	NR	NR	192	72	21.5(11.4–40)	12
Moderate	R	R	263	285	7.4(4–13)	6.6
Moderate	R	NR	487	799	4.9(2.7–8.8)	5.3
Moderate	NR	R	72	157	3.7(1.9–7)	4.7
Moderate	NR	NR	165	363	3.6(2–6.7)	3.7
Low	R	R	13	87	1.2(0.5–2.7)	2.2
Low	R	NR	56	218	2.07(1.08–3.9)	1.7
Low	NR	R	5	37	1.09(0.3–3.3)	1.5
Low	NR	NR	13	105	1 (ref)	1.2

a “R”genotype at given locus associated with risk; “NR”, genotype at given locus not associated with risk;

b Odds ratio vs single reference group without risk genotype at any of the four loci.

## Discussion

The present study is a comprehensive evaluation of the joint effects of the three most well established T1D susceptibility genes in a case-control data set from a Caucasian French population, to assess the joint genetic risk of developing T1D based on the genotype variation at these loci. When the joint risks conferred by all susceptible loci and all non-susceptible loci were evaluated for all three genes, the absolute risk values were 11.5% and 0.02%, respectively. Our results are in agreement with some of the previous studies, confirming that HLA-DRB1 is a more relevant gene of susceptibility compared to INS and PTPN22 and proved, using the BN, that the INS and PTPN22 genotypes marginally influence T1D risk in all different HLA-DRB1 genotype risk categories [29]–[31].

Confirming earlier observations about the heterogeneity in the relative effects of INS [32], [33], we also found evidence that the INS predisposing genotype is significantly less frequent in high-risk HLA-DRB1 genotype positive patients than in those with moderate and low-risk HLA-DRB1 categories. Moreover, also PTPN22 susceptibility alleles conferred, albeit less than INS gene, a higher risk for T1D, both when compared with absolute risk in the general population and when associated with the HLA-DRB1 gene. The relative risk conferred by PTPN22 was stronger in the lower-risk HLA categories than in the high risk HLA category. On the other hand, the protective effect of non-susceptibility genotypes was stronger if INS rather than PTPN22 gene were considered for all of the HLA risk classes. The joint risks assessed in this study were consistent with findings in literature [29], [34]. Motzo et al. studied the joint effect on T1D onset of HLA and INS genes, in a case-control Sardinian cohort, whereas Bjornvold et al. analyzed a sample of case-control subjects under the age of 15 years with the aim of assessing the joint effect of the four main T1D susceptibility genes. Both studies used a T1D prevalence value of 0.4% and classified HLA and INS alleles in risk categories as we did in our study.

Moreover, our study classified T1D risk on the basis of HLA-DRB1, INS and PTPN22 gene combinations in a large group of French Caucasian families. When BN was implemented, the prevalence value of 6% was considered for its training, as confirmed from data in the literature for the T1D risk in siblings [13]. When the relationship between HLA, INS and PTPN22 genotypes was evaluated separately, a significant association between genes and T1D was found (0.003 for HLA, p = 1*10^−3^ for INS gene and p = 3*10^−4^ for PTPN22). Considering the specific genetic compositions in patients and controls from family group, the marked risk in the offspring carrying DRB1*03/*4 was consistent with the high prevalence of this genotype found in our data set (37% and 12% in patients and controls, respectively). When proportions of high risk HLA genotype of family data set were compared with the values present in case-control data set analyzed earlier (30% and 2.7% for patients and controls, respectively), a significant difference was found. Moreover, the analysis of INS and PTPN22 genotypes was done in order to determine whether their addition to HLA genotypes might improve T1D disease prediction. We stratified individual HLA-DRB1 genotypes conferring different risks for T1D confirming the main contribution of the HLA-DRB1 locus to T1D risk and demonstrated that the INS and PTPN22 genes provided only a marginal additional risk for T1D in subjects carrying the high, moderate and low risk HLA-DRB1 genotypes. Furthermore, our results showed that, when HLA and INS genes were considered in patients and controls, a significant heterogeneity in the distribution of the INS genotype (susceptibility/non-susceptibility), according to the HLA risk classes, was found.

Our study showed that a feasible and accurate risk assessment can be performed by applying the BN method. Here the effects of only three genes were evaluated and compared, but the BN method is able to analyze a larger amount of variables with different risk categories for each variable, at the same time. This feature could be crucial in the study of multifactorial diseases, where the triggers involved in the complex mechanisms underlying disease pathophysiology are multiple. Studies in different populations and ethnic groups have indicated some heterogeneity in HLA-associated risk of T1D and it is also possible that gene–gene interactions may vary across populations. Therefore, genes could play a different role depending on the population in which data are collected [36]. Thus, a geographical stratification of T1D risk is essential because of potentially different mechanisms of gene-environment and gene-gene interaction in triggering the disease in different countries. Furthermore, increasing the number of susceptibility loci considered simultaneously, increases the predictive value for the disease. The downside is that the proportion of the population simultaneously carrying multiple risk genotypes becomes minute and that even with relatively large data sets, as in our study, the absolute risk estimate becomes imprecise. The high-risk HLA-DRB1 genotype is carried by 1–2% of the control population and confers a very high risk of disease. Moreover, in our data set, as previously reported [34], [35], only a small proportion of the population (included T1D cases) simultaneously carries the HLA-DRB1 and multiple non-HLA susceptibility genotypes.

By introducing “prior” knowledge from the literature, we can also analyze *small* data sets while maintaining accuracy. In our study, due to the BN approach, a small sample consisting of 73 control subjects was analyzed and the training results matched with the findings in the literature. Prevalence value was used here as prior and, based on that, the network was able to learn the correct rate of genotype combinations characterizing both the general population and patients group and to elaborate data giving coherent results as discussed earlier. Despite to the odds ratio parameter, BN analysis was not affected by lack of data about control subjects with specific genetic combinations (high risk HLA, INS and PTPN22 risk genotypes; high risk HLA, INS and PTPN22 non-susceptibility genotypes and high risk HLA, INS non-susceptibility genotypes and PTPN22 susceptibility genotypes).

In conclusion, the present study represents, to the best of our knowledge, the first study based on both case-control and familiar data sets, showing the joint effect of HLA, INS and PTPN22 in T1D in a Caucasian population with a wide range of age at T1D onset, generalizing previous findings regarding data sets consisting of patients and controls <15 years by Bjørnvold M. et al. [34]. Our results showed that BN represents an alternative way to assess the joint risk to develop T1D by considering different disease genetic markers at once. Although no preventive intervention is available for T1D today, prediction of the disease is an important part of prevention strategies, both for recruitment of participants and for the identification of target populations for future preventive interventions. Understanding the joint effect of the established T1D susceptibility genes will enhance this possibility.
